# Prediction of medical admissions after psychiatric inpatient hospitalization in bipolar disorder: a retrospective cohort study

**DOI:** 10.3389/fpsyt.2024.1435199

**Published:** 2024-09-03

**Authors:** Alessandro Miola, Michele De Prisco, Marialaura Lussignoli, Nicola Meda, Elisa Dughiero, Riccardo Costa, Nicolas A. Nunez, Michele Fornaro, Marin Veldic, Mark A. Frye, Eduard Vieta, Marco Solmi, Joaquim Radua, Fabio Sambataro

**Affiliations:** ^1^ Department of Neuroscience, University of Padova, Padua, Italy; ^2^ Department of Psychiatry and Psychology, Mayo Clinic, Rochester, MN, United States; ^3^ Bipolar and Depressive Disorders Unit, Hospital Clinic de Barcelona, Barcelona, Spain; ^4^ Institut d’Investigacions Biomediques August Pi i Sunyer (IDIBAPS), University of Barcelona, Barcelona, Spain; ^5^ Centro de Investigación Biomédica en Red de Salud Mental (CIBERSAM), Instituto de Salud Carlos III, Madrid, Spain; ^6^ Department of Medicine, Faculty of Medicine and Health Sciences, Institute of Neurosciences (UBNeuro), University of Barcelona (UB), Barcelona, Spain; ^7^ Departament de Medicina, Facultat de Medicina i Ciències de la Salut, Universitat de Barcelona (UB), Barcelona, Spain; ^8^ Department of Psychiatry, University of Utah, Salt Lake City, UT, United States; ^9^ Department of Psychiatry, Federico II University of Naples, Naples, Italy; ^10^ SCIENCES lab, Department of Psychiatry, University of Ottawa, Ottawa, ON, Canada; ^11^ Department of Mental Health, The Ottawa Hospital, Ottawa, ON, Canada; ^12^ Ottawa Hospital Research Institute (OHRI) Clinical Epidemiology Program University of Ottawa, Ottawa, ON, Canada; ^13^ School of Epidemiology and Public Health, Faculty of Medicine, University of Ottawa, Ottawa, ON, Canada; ^14^ Department of Child and Adolescent Psychiatry, Charité Universitätsmedizin, Berlin, Germany

**Keywords:** bipolar disorder, comorbidity, premature mortality, general medicine admission, machine learning, clinical decision-making

## Abstract

**Objective:**

Bipolar Disorder (BD) is a severe mental illness associated with high rates of general medical comorbidity, reduced life expectancy, and premature mortality. Although BD has been associated with high medical hospitalization, the factors that contribute to this risk remain largely unexplored. We used baseline medical and psychiatric records to develop a supervised machine learning model to predict general medical admissions after discharge from psychiatric hospitalization.

**Methods:**

In this retrospective three-year cohort study of 71 patients diagnosed with BD (mean age=52.19 years, females=56.33%), lasso regression models combining medical and psychiatric records, as well as those using them separately, were fitted and their predictive power was estimated using a leave-one-out cross-validation procedure.

**Results:**

The proportion of medical admissions in patients with BD was higher compared with age- and sex-matched hospitalizations in the same region (25.4% vs. 8.48%). The lasso model fairly accurately predicted the outcome (area under the curve [AUC]=69.5%, 95%C.I.=55–84.1; sensitivity=61.1%, specificity=75.5%, balanced accuracy=68.3%). Notably, pre-existing cardiovascular, neurological, or osteomuscular diseases collectively accounted for more than 90% of the influence on the model. The accuracy of the model based on medical records was slightly inferior (AUC=68.7%, 95%C.I. = 54.6-82.9), while that of the model based on psychiatric records only was below chance (AUC=61.8%, 95%C.I.=46.2–77.4).

**Conclusion:**

Our findings support the need to monitor medical comorbidities during clinical decision-making to tailor and implement effective preventive measures in people with BD. Further research with larger sample sizes and prospective cohorts is warranted to replicate these findings and validate the predictive model.

## 1 Introduction

Bipolar Disorder (BD) is a severe mental illness that causes significant disability worldwide (1, 2). BD is associated with strikingly high rates of general medical comorbidities (above 90% of cases endorse at least one comorbidity), especially cardiovascular, metabolic, endocrine, respiratory, and musculoskeletal diseases (3, 4). Older age, higher body mass index (BMI), and longer duration of the illness increase the burden of general medical comorbidities among people with a primary diagnosis of BD (5–7), affecting both the course and the treatment outcomes of either of the associated conditions (8).

Accruing evidence underscores the burden of cardiometabolic disease in BD, as highlighted by the higher rates of obesity (46% vs. 37%, Odds Ratio=1.62 [95%C.I. = 1.22–2.15]), elevated systolic blood pressure (35% vs. 19%, OR=2.18 [95%C.I. = 1.55–3.06]) and elevated serum triglyceride levels (37% vs. 26%, OR=1.58 [95%C.I. = 1.13–2.20]) in patients with BD compared to the matched controls (9). Additionally, patients with BD have an increased risk of fatal or non-fatal myocardial infarction or stroke (hazard ratio [HR]=1.54 [95%C.I. = 1.02–2.33]) (10) and an increased risk of major adverse cardiac events (MACE; HR=1.66 [95%C.I. = 1.17–2.28]) (11, 12).

Several factors may contribute to poor physical health among patients with BD, including genetic vulnerability (13, 14), atypical depressive symptoms (15, 16), abnormal feeding behaviors and eating disorders such as binge eating (17, 18), unhealthy lifestyle with reduced physical activity (19, 20), poorer quality diet (21), tobacco smoking (22) and other substance use disorders (23), as well as adverse effects of psychotropic pharmacotherapy, such as weight gain and metabolic syndrome (24–26). Of note, BD is associated with an increased all-cause, suicide, and natural cause-related mortality (27) that is estimated to be twice higher compared to the general population (28) and with a shorter life expectancy, with a recent meta-analysis estimating an average of 13 years of potential life lost compared to the general population (29). Patients with BD die prematurely from multiple causes, including cardiovascular disease, diabetes, chronic obstructive pulmonary disease, influenza or pneumonia, unintentional injuries, and suicide (27, 28, 30). The medical burden in BD is also associated with significant costs, both direct – hospital admissions, use of medical resources – and indirect – loss of work and productivity (31–34).

In addition, previous evidence highlights that discharged psychiatric inpatients are at increased risk of serious adverse outcomes, including all-cause mortality (35). Notably, BD patients are more susceptible to medical hospitalization (36). This underscores the critical need to delve into the complex dynamics of post-hospitalization care for BD patients, emphasizing the imperative for tailored interventions and a comprehensive healthcare approach. Although high hospitalization rates, healthcare costs (37), and increased medical comorbidity are well known in BD patients (4, 14), the factors associated with their increased risk of medical admission after discharge from psychiatric units remain largely unexplored.

In this retrospective observational cohort study, we aimed to identify predictors of medical admissions after discharge from psychiatric hospitalization of people with a primary diagnosis of BD. Given the large number of predictors, we used a machine learning approach that allows the estimation of predictive models from high-dimensional data and automates feature selection while mitigating the bias introduced by manually selecting subsets of potential predictors typically required in traditional regression models (38–40). Compared to standard stepwise regression, this approach provides a more “parsimonious” model, thus avoiding overfitting when multiple predictors are tested.

## 2 Materials and methods

### 2.1 Study cohort

The present retrospective observational cohort study documented inpatients admitted to the psychiatric ward of Padova University Hospital (Padua, Italy) from July 2017 to July 2019, with an established diagnosis of BD according to DSM-IV (i.e., type I, II, or not otherwise specified [NOS] BD, but not cyclothymia). All patients included in this study previously signed a written informed consent for the general use of their data for research purposes anonymously and in aggregate form. In accordance with our local Internal Review Board, the passive review of medical records for this retrospective and naturalistic research study did not require patients to provide further informed consent. This study was carried out in accordance with the guidelines of the Declaration of Helsinki of 1975.

### 2.2 Assessed variables

Demographic data (i.e., age and sex), anthropometric measures (weight and height), electrocardiograms (ECG), routine blood tests (i.e., cholesterol, glucose, hemoglobin, platelets, potassium, triglycerides, TSH levels and white blood cells count), characteristics of BD (age at onset, and polarity of the index episode, presence of psychotic symptoms during inpatient observation), number of comorbid mental disorders (other than BD), number and type of medical comorbidities (i.e., cancer, cardiovascular, diabetes, hypertension, dyslipidemia, hematological, endocrine, gastrointestinal, gynecological, infectious, renal, hepatic, neurological, osteomuscolar, respiratory, and thyroid disorders), number and type of prescribed medications (i.e., psychiatric: antipsychotics, antidepressants, mood stabilizers, and other psychotropic medications; and non-psychiatric medications), administration of long-acting injectable antipsychotics before admission were gathered for the consecutively admitted patients with BD. A detailed list of the variables collected is available in the **Supplementary Material**, **Supplementary Table 1**. We followed up with participants for three years and recorded the first and each subsequent hospitalization for non-psychiatric reasons until the end of the follow-up or the participant’s death.

### 2.3 Statistical analyses

For descriptive statistics, categorical variables were summarized by frequency, proportions, and percentages, while mean, median, interquartile range (IQR), standard deviation (SD), and confidence intervals (95%CIs) were used for continuous variables. The proportions of medical admissions for patients with BD recruited in the current study were compared with age- and sex-matched hospitalizations in the general population of the same region (Veneto, Italy) with a chi-squared test (χ^2^) (41). Using variables collected during psychiatric hospitalization (baseline), we attempted to create predictive models of subsequent admission to any general medical ward within the next three years of follow-up. The first model was designed to predict the occurrence of hospitalization in the general medical ward using any collected measures (i.e., biochemical, clinical, ECG-related, psychiatric, and sociodemographic). The second model considered the same outcome and used psychiatric and sociodemographic variables only as predictors. Finally, the third model used medical, including biochemical, clinical, ECG-related, and sociodemographic variables only as predictors. First, we used the “least absolute shrinkage and selection operator” (LASSO) logistic and normal regression models to impute the missing values 20 times (42). This supervised machine learning technique handles the problem of overfitting by including a penalty term that shrinks coefficient estimates toward zero and removes non-critical characteristics from the model. Second, we used another LASSO logistic regression to predict whether a participant would be admitted to any general medical ward during follow-up. We used a leave-one-out cross-validation procedure to subdivide the original sample into two non-overlapping, independent subsamples: the training subsample (in which we fitted the models to impute and the model to predict) and the test subsample (used to check whether the model predicted correctly). We calculated the area under the curve (AUC) and the 95% CIs, sensitivity, specificity, and balanced accuracy (BAC) to estimate the model’s performance. For each variable, the importance scores were obtained by normalizing the absolute values of their coefficients across multiple models and imputations and thresholded as ‘relevant’ using a cut-off of at least 1%. This represents the relative importance or contribution of each predictor to the model. A receiver operating characteristic (ROC) plot was used to show the relationship between sensitivity and specificity for each prediction model. Initially, we aimed to predict the number of hospitalizations using a Poisson LASSO regression but the small number of patients with >1 admission (n=7) did not allow a proper fit of the model. We imputed the missing data, trained the machine learning models, and validated them using R version 4.1.2 (43) and the R package *glmnet* (44).

## 3 Results

Seventy-one patients were eligible for inclusion, consented to participate, and completed the three-year follow-up study (see **Table 1**). Briefly, 45 participants were diagnosed with BD-I, 13 with BD-II, and 13 with BD-NOS. The mean age was 52.19 years old (SD=14.29); 40 participants were female (56.33%). The prevalent polarity was the only baseline characteristic statistically significantly different across different types of BD. Patients with BD-II or BD-NOS had a higher number of depressive episodes than hypomanic episodes. In contrast, patients with BD-I prevalent polarity were more frequently manic (48.9%) than depressive (26.7%) or undetermined (20%). The total number of BD patients admitted to a medical ward was 18: 13 BD-I (with six patients admitted twice or more), 2 BD-II, and 3 BD-NOS (with one admitted three times). The proportion of medical admissions at three-year follow-up in our sample (25.4%) was significantly higher compared with age- and sex-matched hospitalizations (8.48%) in the same region (p<0.001). Five patients (7%) died during follow-up; four for medical reasons (cancer, COVID-19, or unknown) and one for suicide.

**Table 1:** Sample characteristics.

	BD-I	BD-II	BD-NOS	p-value (F or χ²)
**Sample size**	45	13	13	
**Age (mean (SD))**	52.60 (15.06)	51.46 (15.22)	51.54 (11.25)	n.s
**Length of first hospitalization (median [IQR])**	15.00 [8.00, 22.00]	14.00 [7.00, 17.00]	10.00 [6.00, 15.00]	n.s.
**Gender (N (Female %))**	23 (51.1)	8 (61.5)	9 (69.2)	
Mood stabilizer at first discharge (%)				
No.	15 (33.3)	5 (38.5)	5 (38.5)	n.s.
Yes	30 (66.7)	8 (61.5)	8 (61.5)	
**No. of comorbidities (median [IQR])**	1.00 [0.00, 2.00]	1.00 [0.00, 2.00]	0.00 [0.00, 2.00]	n.s.
**No. of medications prescribed (median [IQR])**	3.00 [2.00, 5.00]	3.00 [3.00, 4.00]	3.00 [2.00, 4.00]	n.s.
Age at Onset < 18 (%)				
Not available	9 (20.0)	3 (23.1)	0 (0.0)	n.s.
No	35 (77.8)	8 (61.5)	13 (100.0)	
Yes	1 (2.2)	2 (15.4)	0 (0.0)	
Index Episode (%)				
Depressive	21 (46.7)	10 (76.9)	6 (46.2)	n.s.
[Hypo]manic	16 (35.6)	0 (0.0)	2 (15.4)	
Not available	8 (17.8)	3 (23.1)	5 (38.5)	
Long-acting Injection (%)				
No	30 (66.7)	11 (84.6)	12 (92.3)	n.s.
Yes	15 (33.3)	2 (15.4)	1 (7.7)	
Prevalent Polarity (%)				
Depressive	12 (26.7)	11 (84.6)	7 (53.8)	<0.001
Manic	22 (48.9)	0 (0.0)	0 (0.0)	
Mixed	2 (4.4)	2 (15.4)	4 (30.8)	
None	9 (20.0)	0 (0.0)	2 (15.4)	
**No. Psych Admissions (median [IQR])**	2.00 [1.00, 3.00]	2.00 [1.00, 2.00]	2.00 [1.00, 2.00]	n.s.
**No. Med Admissions (median [IQR])**	0.00 [0.00, 1.00]	0.00 [0.00, 0.00]	0.00 [0.00, 0.00]	n.s.
**No. Psych Admissions (Total)**	45	13	13	
**No. Med Admissions (Total)**	13	2	3	
No. deaths				
Suicide	0	0	1	
Other causes (unknown, lymphoma, COVID-19)	4	0	0	

BD-I, Bipolar Disorder Type I; BD-II, Bipolar Disorder Type II; BD-NOS, Bipolar Disorder – Not Otherwise Specified; IQR, interquartile range; n.s., not significant; SD, standard deviation.

### 3.1 Prediction of hospitalization in any general medical ward using all variables (global model)

**Figure 1:**
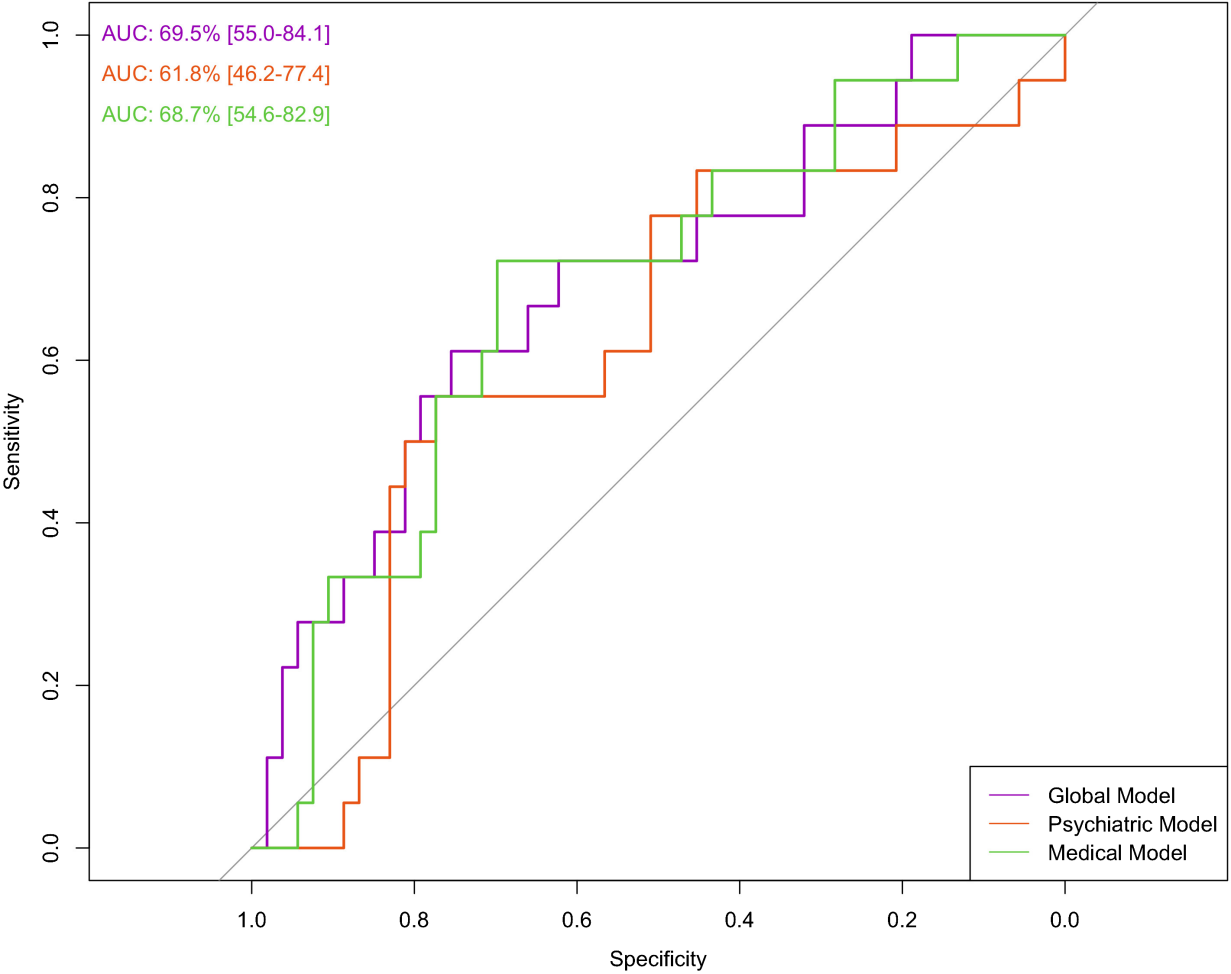
Prediction of hospitalization in any general medical ward using all variables (global model), psychiatric variables (psychiatric model), and medical variables (medical model). The ROC curve shows the relationship between sensitivity and specificity for each prediction model.

**Figure 2:**
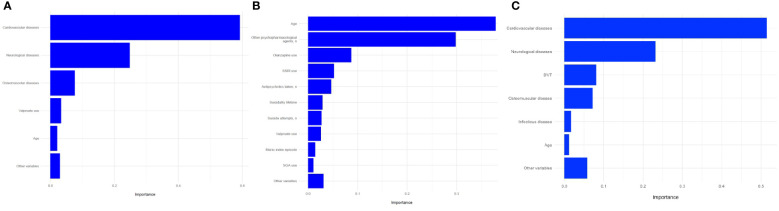
Prediction of hospitalization in any general medical ward using **(A)** all variables (global model); **(B)** psychiatric variables (psychiatric model); **(C)** medical variables (medical model); variable importance. SSRI, Selective serotonin reuptake inhibitor; SGA, Second-generation antipsychotic; DVT, deep vein thrombosis.

In this model, we used 109 baseline variables (i.e., 10 biochemical, 31 clinical, 11 ECG-related, 55 psychiatric, and 2 sociodemographic) to predict the occurrence of medical admission during the three years of follow-up. The model was able to predict the outcome (AUC=69.5%, 95%C.I. = 55-84.1). The sensitivity was 61.1% and the specificity 75.5%, resulting in a BAC=68.3%. A ROC plot is presented in **Figure 1**. The most relevant variables in the model were cardiovascular disease (importance=59.2%); neurological diseases (importance=24.8%); osteomuscular diseases (importance=7.6%); valproate (importance=3.3%); and age (importance=2.1%) (**Figure 2A**). The sum of the importance of all remaining variables was 3%.

### 3.2 Prediction of hospitalization in any general medical ward using sociodemographic and psychiatric variables (psychiatric model)

In this model, we used psychiatric (n=55) and sociodemographic (n=2) baseline variables only. The model did not predict the outcome (AUC= 61.8%, 95%C.I. = 46.2-77.4). The sensitivity was 55.5%, and the specificity 66.0%, resulting in a BAC of 60.8%. A ROC curve is presented in **Figure 1**. The most relevant variables of the model were age (importance=37.9%), number of other psychopharmacological agents (importance=29.8%), olanzapine use (importance=8.7%), SSRI use (importance=5.2%), number of antipsychotics (importance=4.7%), suicidality lifetime (importance=2.9%), number of suicide attempts (importance=2.7%), valproate use (importance=2.6%), manic index episode (importance=1.4%), and second-generation antipsychotic use (importance=1%) (**Figure 2B**). The sum of the importance of all remaining variables was 3.1%.

### 3.3 Prediction of hospitalization in any general medical ward using sociodemographic, biochemical, clinical, and ECG-related variables (medical model)

We used baseline variables related to medical factors in this model (i.e., 10 biochemical, 31 clinical, 11 ECG-related, and 2 sociodemographic). The model was able to predict the outcome (AUC=68.7%, 95%C.I. = 54.6-82.9). Sensitivity was 55.5%, and specificity 71.6%, resulting in a BAC of 63.6%. A ROC curve is presented in **Figure 1**. The most relevant variables in the model were cardiovascular disease (importance=51.5%), neurological disease (importance=23.2%), deep vein thrombosis (importance=8%), osteomuscular disease (importance=7.1%), infectious disease (importance=1.7%), age (importance=1.2%) (**Figure 2C**). The sum of the importance of all remaining variables was 5.8%.

## 4 Discussion

The current three-year retrospective cohort study of 71 patients diagnosed with BD confirmed an increased susceptibility to medical hospitalization aligning with previous reports (36, 45, 46). Using baseline biochemical, clinical, ECG-related, psychiatric, and sociodemographic variables, we developed three supervised machine learning models and two out of three were able to predict general medical admission after a psychiatric exacerbation.

The global model included all variables and showed a fair predictive performance, being able to discriminate those undergoing a medical hospitalization during follow-up with a BAC of 68.3%. The most important variables were the co-occurrence of cardiovascular, neurological, or osteomuscular diseases, which together accounted for more than 90% of the influence on the model.

Again, the medical model, which included all the variables of the global model except the psychiatric ones, was able to predict the outcome, resulting in a BAC of 63.6%. These findings highlight the critical importance of recognizing and addressing medical comorbidity as a significant issue in patients with BD. Indeed, physical health conditions are more frequent in BD compared to the general population, with a two-fold increase in mortality, even after adjusting for age and other sociodemographic factors (28). Additionally, compared to other psychiatric conditions such as schizophrenia spectrum disorders, patients with BD were more likely to have at least one physical comorbidity (47), with specific differences in cardiovascular, nutritional, and metabolic diseases (48). The reasons why these aspects can strongly influence subsequent hospitalizations in people diagnosed with BD may lie in the fact that people with severe mental illness, commonly receive poorer quality of care for physical comorbidities due to several barriers related to patient, treatment, physician, and service (49–51). Therefore, the importance of these variables in the models calls for greater attention to their monitoring, which often appears to be considered of minor relevance (52), to ensure better follow-up of patients and potentially reduce the risk of hospitalizations. Although not as important as the variables discussed so far, the use of valproate appeared to have a modest effect on the outcome of interest. Valproate treatment has been associated with a higher incidence of side effects such as tremors (53), abdominal pain, vomiting (54), dizziness, somnolence (55), or weight gain (55, 56) compared with placebo, teratogenic risk (57), and with increased somnolence or nausea compared with other drugs such as lithium or olanzapine (55, 58). Considering this safety profile, which could worsen a poor physical condition, our results urge caution in the use of this drug. In this light, and also considering the impact of neurological disorders on hospitalizations, the neuroprotective profile associated with the use of lithium seems to be a recommended choice to reduce the physical burden of these patients (59–61).

The psychiatric model included only psychiatric and sociodemographic variables and did not significantly discriminate against those admitted to a general medical ward during follow-up. Notably, while psychopharmacological therapies can have an impact on the physical health of the individual (24), they do not predict a medical hospitalization. Although the reduced model was not significant, age showed an impact of about 40% on overall prediction, thus suggesting a need for greater clinical attention to older patients with BD (62).

Furthermore, in our sample, patients with BD-I were more likely to be admitted to the medical wards (28.9%) than those with BD-II (15.4%) or BD-NOS (23.1%). These findings are consistent with previous reports showing that patients with BD-I had a higher probability of presenting at least one co-occurring chronic medical disorder diagnosed by the physician than patients who screened negative for a manic episode (64.3 vs. 48.5%) (63). Thus, our findings could also be related to the association between chronic medical disorders and the severe course of BD (63). Interestingly, among the psychiatric variables that contributed to the model, current drug treatments, including the type and number of medications, played an important role. Although the overall model did not significantly predict the outcome, we can speculate that specific medications could contribute to an increased risk of medical comorbidities during follow-up (i.e., antipsychotic medications) and cardiovascular and metabolic diseases (12). On the other hand, non-adherence to pharmacological treatment can also increase the risk of all-cause rehospitalization (64, 65). In our sample, during the 3-year retrospective follow-up, we found a mortality rate of 7%, which is consistent with the epidemiological scenarios of the COVID-19 pandemic (66) and the excess mortality rate it caused (67).

Although large evidence in BD has identified being uninsured, the number of previous psychiatric hospitalizations, a younger age, experiencing depressive episodes characterized by prominent neurovegetative features, the number of previous mood episodes, or having lower global functioning and greater severity at discharge as the risk factors for psychiatric readmission (68–72), the predictors associated with a greater probability of general medical hospitalization are less studied and poorly appraised. In our model, although psychiatric and sociodemographic variables contributed to the risk of hospitalization in the global model, they were unable to predict the outcome when the analysis included only these variables. Overall, our findings support the idea that the burden in BD is due not only to sociodemographic and psychiatric conditions but also to general medical problems. They influence specifically medical admission but could also contribute to psychiatric exacerbation. Previous evidence synthesis conducted by Šprah and colleagues (73) supports the hypothesis that patients with mental disorders are at increased risk of psychiatric readmission if they have a co-occurring medical condition, and the same result is confirmed in BD (40, 74).

The current findings should be interpreted in light of some limitations. First, the small sample size did not allow the analysis of other outcomes (such as mortality and survival analysis) and the stratification for the types of BD (75). Larger cohorts are needed to validate the predictive models and enhance their robustness. Second, the retrospective study design may introduce inherent limitations, such as information bias and missing data, which, together with the lack of nested control for BD patients who would not be admitted to a general medical ward, limit our ability to establish causality in the current findings. Third, the regional basis of the study may limit the generalizability of our findings to other regions or healthcare settings. Fourth, the lack of external validation did not allow us to assess a model’s performance and reproducibility. Finally, the exclusion of patients with cyclothymia may limit the comprehensiveness of the current study in representing the entire BD spectrum. Although we decided to exclude patients with cyclothymia to reduce clinical heterogeneity and focus on more severe and homogeneous BD phenotypes, including all BD spectrum disorders could have provided a more complete understanding of the medical comorbidities and hospitalization risks associated with BD.

In conclusion, to our knowledge, this is the first study that specifically aimed to investigate predictors associated with medical admissions after discharge from psychiatric hospitalization in a cohort of BD, and although risk factors for hospitalization in psychiatric settings are well known, factors influencing medical hospitalization have not been studied extensively. Although the model we propose to describe medical hospitalization in patients with BD is in the development stage, the current study raises the need to further examine and carefully monitor medical comorbidities during clinical judgments in people with BD. Future research should employ prospective study designs with larger, multicenter cohorts to enhance the generalizability of our findings and provide more robust evidence regarding causality while better controlling for potential confounders. More prospective studies are needed to replicate and validate this model, as well as to quantify the risk of medical admission after discharge from the psychiatric ward in this vulnerable population to tailor effective preventive measures.

## Data Availability

The raw data supporting the conclusions of this article will be made available by the authors, without undue reservation.
