# Supplementary material for: Prediction of medical admissions after psychiatric inpatient hospitalization in bipolar disorder: a retrospective cohort study
Source: Front Psychiatry. 2024 Sep 3;15:1435199. doi: 10.3389/fpsyt.2024.1435199 (PMC11406175; doi:10.3389/fpsyt.2024.1435199)
Supplement: Supplementary file 1 [file Table1.docx]

Supplementary Material

**Prediction of Medical Admissions after Psychiatric Inpatient Hospitalization in Bipolar Disorder: a Retrospective Cohort Study**

Alessandro Miola^1,2^, Michele De Prisco^3,4,5,6,7^, Marialaura Lussignoli^1^, Nicola Meda^1^, Elisa Dughiero^1^, Riccardo Costa^1^, Nicolas A. Nunez^2,8^, Michele Fornaro^9^, Marin Veldic^2^, Mark A. Frye^2^, Eduard Vieta ^3,4,5,6,7^, Marco Solmi ^10,11,12,13,14^, Joaquim Radua ^4,5,7^, Fabio Sambataro^1*^

^1^Department of Neuroscience, University of Padova, Padua, Italy;

^2^Department Psychiatry and Psychology, Mayo Clinic. Rochester, MN, USA;

^3^Bipolar and Depressive Disorders Unit, Hospital Clinic de Barcelona, Barcelona, Spain;

^4^Institut d'Investigacions Biomediques August Pi i Sunyer (IDIBAPS), University of Barcelona, Barcelona, Spain;

^5^Centro de Investigación Biomédica en Red de Salud Mental (CIBERSAM), Instituto de Salud Carlos III, Madrid, Spain;

^6^Institute of Neurosciences (UBNeuro), Barcelona, Spain;

^7^Departament de Medicina, Facultat de Medicina i Ciències de la Salut, Universitat de Barcelona (UB), Barcelona, Spain;

^8^Department of Psychiatry, University of Utah, Salt Lake City, Utah. USA

^9^Department of Psychiatry, Federico II University of Naples, Italy.

^10^SCIENCES lab, Department of Psychiatry, University of Ottawa, Ontario, Canada;

^11^Department of Mental Health, The Ottawa Hospital, Ontario, Canada;

^12^Ottawa Hospital Research Institute (OHRI) Clinical Epidemiology Program University of Ottawa Ontario;

^13^School of Epidemiology and Public Health, Faculty of Medicine, University of Ottawa, Ottawa, Canada;

^14^Department of Child and Adolescent Psychiatry, Charité Universitätsmedizin, Berlin, Germany;

*** Correspondence:**Fabio Sambataro, MD, PhD

Department of Neuroscience (DNS), University of Padova, Italy

Via Giustiniani 5, Padova

Tel:+390498211980

e-mail: fabio.sambataro@unipd.it

**Table S1. Complete list of the variables included in the overall model (n=109)**

| **Category** | **Variable** |
| --- | --- |
| Biochemical | Cholesterol HDL |
| Biochemical | Cholesterol LDL |
| Biochemical | Glucose |
| Biochemical | Hemoglobin |
| Biochemical | Platelets |
| Biochemical | Potassium |
| Biochemical | Total cholesterol |
| Biochemical | Triglycerides |
| Biochemical | TSH |
| Biochemical | White blood cells |
| Clinical | Autoimmune diseases |
| Clinical | Benign prostatic hypertrophia |
| Clinical | BMI at admission |
| Clinical | BMI at discharge |
| Clinical | BMI>30 at admission |
| Clinical | Cancer |
| Clinical | Cardiovascular diseases |
| Clinical | Diabetes |
| Clinical | Diastolic blood pressure |
| Clinical | Deep vein thrombosis |
| Clinical | Dyslipidemia |
| Clinical | Hematological diseases |
| Clinical | Endocrine diseases |
| Clinical | Gastrointestinal diseases |
| Clinical | General medical comorbidity |
| Clinical | GFR |
| Clinical | Gynecological diseases |
| Clinical | Height |
| Clinical | Hypertension |
| Clinical | Infectious diseases |
| Clinical | Kidney diseases |
| Clinical | Liver diseases |
| Clinical | Neurological diseases |
| Clinical | Number of general medical comorbidities |
| Clinical | Number of medications |
| Clinical | Osteomuscolar diseases |
| Clinical | Respiratory diseases |
| Clinical | Systolic blood pressure |
| Clinical | Thyroid disorders |
| Clinical | Weight at admission |
| Clinical | Weight at discharge |
| ECG-related | Any issue at ECG (dichotomous) |
| ECG-related | Heart ratio |
| ECG-related | Other issues at ECG (dichotomous) |
| ECG-related | PR length |
| ECG-related | QRS length |
| ECG-related | QTC length |
| ECG-related | QTC length (dichotomous) |
| ECG-related | Repolarization issues at ECG (dichotomous) |
| ECG-related | Rhythm and repolarization issues at ECG (dichotomous) |
| ECG-related | Rhythm issues at ECG (dichotomous) |
| ECG-related | Structural issues at ECG (dichotomous) |
| Psychiatric | Age at onset of BD before 18 years old |
| Psychiatric | Amisulpride use |
| Psychiatric | Antidepressants use |
| Psychiatric | Antipsychotics LAI use |
| Psychiatric | Antipsychotics use |
| Psychiatric | Any psychiatric drug use |
| Psychiatric | Aripiprazole use |
| Psychiatric | Asenapine use |
| Psychiatric | BD type |
| Psychiatric | Chlorpromazine equivalent |
| Psychiatric | Chlorpromazine use |
| Psychiatric | Clotiapine use |
| Psychiatric | Clozapine use |
| Psychiatric | Depressive index episode |
| Psychiatric | Depressive predominant polarity |
| Psychiatric | First-generation antipsychotics use |
| Psychiatric | Fluphenazine use |
| Psychiatric | Haloperidol use |
| Psychiatric | Lamotrigine dose |
| Psychiatric | Lamotrigine use |
| Psychiatric | Length of stay |
| Psychiatric | Levomepromazine use |
| Psychiatric | Lithium dose |
| Psychiatric | Lithium use |
| Psychiatric | Lurasidone use |
| Psychiatric | Manic index episode |
| Psychiatric | Manic predominant polarity |
| Psychiatric | Mood stabilizers use |
| Psychiatric | Number of antidepressants taken |
| Psychiatric | Number of antipsychotics taken |
| Psychiatric | Number of mood stabilizers taken |
| Psychiatric | Number of other psychopharmacological agents taken |
| Psychiatric | Number of psychiatric hospitalizations |
| Psychiatric | Number of suicide attempts |
| Psychiatric | Olanzapine use |
| Psychiatric | Other psychopharmacological agents use |
| Psychiatric | Paliperidone use |
| Psychiatric | Perphenazine use |
| Psychiatric | Presence of agitation |
| Psychiatric | Presence of psychotic symptoms |
| Psychiatric | Promazine use |
| Psychiatric | Psychiatric comorbidity |
| Psychiatric | Quetiapine use |
| Psychiatric | Risperidone use |
| Psychiatric | Second-generation antipsychotics use |
| Psychiatric | SNRI use |
| Psychiatric | SSRI use |
| Psychiatric | Substance use |
| Psychiatric | Suicidality lifetime |
| Psychiatric | TCA use |
| Psychiatric | Unclear predominant polarity |
| Psychiatric | Valproate dose |
| Psychiatric | Valproate use |
| Psychiatric | Ziprasidone use |
| Psychiatric | Zuclopenthixol use |
| Sociodemographic | Age |
| Sociodemographic | Sex |

**Legend.** HDL: High-density lipoprotein; LDL: Low-density lipoprotein; TSH: Thyroid stimulating hormone; BMI: Body Mass Index; GFR: Glomerular filtration rate; ECG: Electrocardiogram; BD: Bipolar disorder; LAI: Long-acting injectable antipsychotic; SNRI: Serotonin-norepinephrine reuptake inhibitor; SSRI: Selective serotonin reuptake inhibitor; TCA: Tricyclic antidepressant.
